# Structure Analysis of the *MatA* Locus of Sexual Compatibility in the Edible Mushroom *Pleurotus ostreatus*

**DOI:** 10.1134/S1607672923700254

**Published:** 2023-10-13

**Authors:** A. V. Shnyreva, A. A. Shnyreva

**Affiliations:** Moscow State University, Moscow, Russia

**Keywords:** basidiomycetes, homeodomain transcription factors, mating type loci, sexual compatibility

## Abstract

The edible oyster mushroom *Pleurotus ostreatus* is one of the most cultivated species worldwide. Morphogenesis associated with the maturation of fruit bodies is controlled by two unlinked loci of sexual compatibility *matA* and *matB* with multiple alleles (tetrapolar system of sexual compatibility). Quantitative analysis of the alleles of mating compatibility loci in 17 natural isolates collected in the Moscow region was performed in mon–mon (monokaryons–monokaryon) and di–mon (dikaryon–monokaryon) crossings. Four monokaryotic testers strains which were heteroallelic at both mating type loci were obtained for each of the five natural mushroom isolates by using original technique of sterile spore prints on Petri dishes and mon–mon crossing. Twelve natural isolates were crossed via di-mon mating with the four monokaryotic testers M-38. Genetic analysis of the alleles of sexual compatibility loci in 17 natural isolates revealed multiple alleles at both loci: at least ten alleles at *matA* locus and eight alleles at *matB* locus. Structural organization analysis of the *matA* locus was performed in silico for homokaryotic strains PC9 and PC15 based on the whole-genome sequencing data available at DOE Joint Genome Institute. The *matA* locus has an extremely divergent structure: there are one copy of the homeodomain gene *hd1* and one copy of the *hd2* gene in the PC9 strain, whereas the *matA* locus of the PC15 strain is composed by two copies of *hd1.1* and *hd1.2* genes (class HD1 homeodomain proteins) and one copy of *hd2* gene (class HD2 proteins). Comprehensive analysis of amino acid sequences of HD1 and HD2 homeodomain proteins demonstrated that the proteins have a globular structure with the nuclear localization and contain a variable N-terminus and a more conserved DNA-binding domain with a specific conserved motif  WFXNXR in the third ɑ-helix. The results suggest that multiple alleles of the *matA* locus of sexual compatibility in basidiomycete fungi is achieved due to both different copy number of the coding *hd* genes within the locus and the variability of the coding gene sequences.

## INTRODUCTION

The majority of homobasidial fungi have a haploid-dikaryotic life cycle, which is represented by two alternating phases—monokaryotic (haploid) and dikaryotic (functionally diploid). Fruiting bodies can be formed only on the dikaryotic mycelium. In the fertile part of fruiting bodies, haploid basidiospores are formed on basidia, which germinate in haploid monokaryotic mycelium, which is not able to form fruiting bodies. The formation of a fertile (fruit-bearing) dikaryotic mycelium requires the fusion of two sexually compatible haploid mycelia, which are heteroallelic at two unlinked loci (*matA* and *matB*) with multiple alleles (AxBx and AyBy) [1, 2]. Earlier, in natural populations of oyster mushrooms of the genus *Pleurotus* in Moscow and Voronezh regions, we identified 37 alleles of the *matA* locus and 35 alleles of the *matB* locus for the species *P. pulmonarius* and 24 alleles of the *matA* locus and 21 alleles of the *matB* locus for *P. ostreatus* [3, 4]. Multiallelicity of A and B loci was also noted for other species: *P. djamor* (58 *matA* and 231 *matB*) and *P. populinus* (126 *matA* and 354 *matB*) [5, 6]. The *matA* and *matB* loci differ in structure and have different regulatory functions. From the studies performed on the model basidiomycetes *Coprinopsis cinerea* and *Schizophyllum commune*, it is known that the *matA* locus encodes two types of homeodomain transcription factors (HD1 and HD2), while the *matB* locus encodes the genes for pheromones and pheromone receptors [7]. The *matB* locus is studied most comprehensively due to the fact that the pheromone and pheromone receptor genes encoded in it have a conserved structure. The genes of the *matA* locus, in contrast to *matB*, are highly variable in nucleotide sequences, which hampers their study. The structural organization of the *matA* locus was studied in detail on the model fungus *Coprinopsis cinerea*, which has three pairs of genes within this locus; each pair of genes (cassette) encodes two types of homeodomain transcription factors (HD1 and HD2) [8]. However, the structure of the *matA* locus with the complete set of all three pairs of genes (cassettes) can rarely be found in the studied species of basidiomycetes. Within the cassettes of the locus, deletions of one of the *hd* genes are often found, or, conversely, one to several copies of the *hd1* or *hd2* genes can be encoded in one *matA* locus (for example, as in the homobasidiomycete fungi *Flammulina velutipes*, *Lentinula edodes*, *Fomitiporia mediterranea*, *Schizophyllum commune*, and *Hypsizygus marmoreus* [9–12]). As a result of dimerization of two compatible HD1–HD2 proteins from different sexual partners (monokaryons), an active heterodimeric transcription factor is formed, which triggers the expression of genes specific for the dikaryon development, as well as suppresses the specific “monokaryotic” genes functioning in the haploid mycelium.

The aim of the study was to quantify the alleles of the sexual compatibility loci in natural oyster mushroom isolates collected in a limited area and to assess the overall allelic diversity, as well as to analyze in silico the structure of the *matA* sexual compatibility locus of *Pleurotus ostreatus* on the basis of whole-genome sequencing data.

## MATERIALS AND METHODS

### Collection and Cultivation of Natural Isolates of Pleurotus ostreatus

The fruiting bodies of the oyster mushroom (*P. ostreatus*) were collected on the territory of the Zvenigorod Biological Station of Moscow State University, Moscow oblast, in August 2018 from various substrates (aspen, birch, and mountain ash). From the fruiting bodies, 17 mycelial dikaryotic isolates were obtained, which were deposited in the collection of the Department of Mycology and Algology of Moscow State University. Cultivation of strains and crosses were performed on Petri dishes with wort agar (150 mL of beer wort, 850 mL of water, and 20 g of agar) at 25°C in the dark.

### Genetic Analysis of Allele Diversity of Sexual Compatibility Loci in Natural Strains of Pleurotus ostreatus

**Obtaining sterile basidiospore prints.** To perform a genetic analysis of sexual compatibility, we developed a technique for obtaining fruiting bodies and sterile spore imprints directly on Petri dishes. For this purpose, the mycelium was inoculated in the center of the Petri dish and incubated in the dark at 25°C until the surface of the Petri dish was completely overgrown with mycelium (approximately 7–8 days). Then, Petri dishes were placed in a refrigerator at 4°C for 2 days to stimulate fruiting by cold, after which they were incubated upside down at room temperature (23–25°C) and natural light with a day–night change. On average, on the 10–14th day, the appearance of miniature fruiting bodies on the surface of the mycelium and spore imprints of basidiospores on the lid of the Petri dish was noted.

**Obtaining haploid testers of sexual compatibility and quantifying the allelic diversity of loci.** To perform a genetic analysis of the frequency of alleles of sexual compatibility loci in natural strains of *P. ostreatus*, haploid testers of sexual compatibility (mating types) were obtained by the standard procedure (basidiospore seeding from sterile spore imprints on Petri dishes [3]). Quantification of alleles of sexual compatibility factors was performed using the obtained monobasidiospore haploid tester strains heteroallelic at A and B factors of sexual compatibility in mon–mon and di–mon crosses [3]. Mon–mon crosses were performed between monokaryotic testers (four tester strains AxBx, AyBy, AxBy, and AyBx for each natural isolate). Di–mon crosses were performed between the dikaryotic natural strains and four testers of the species *P*. *ostreatus* (strain M-38) from our collection.

### In Silico Analysis of the Structure of the Sexual Compatibility matA Locus

**Analysis of the** ***matA*** **locus structure in species of the genus** ***Pleurotus*.** For structural analysis of the *matA* locus in silico, sequences were taken from electronic databases: sequences of the *matA* locus of *P. ostreatus* monokaryotic strains PC9 and PC15 (Joint Genome Institute, http://jgi.doe.gov/); sequences of *P. djamor* monokaryotic strains RV95/134.104 and RV95/957.30 (GenBank accessions AY462112 and AY462111); and sequences of *P. eryngii* monokaryotic strains CCMSSC00488 and CCMSSC00489 (GenBank accessions HQ595186 and HQ595187). To perform multiple alignments and search for homology between sequences, we used the Dialign 2.2.1 software (http://bibiserv.techfak.uni-bielefeld.de/dialign) [13].

**Analysis of the structure of the** ***matA*** **locus of** ***Pleurotus ostreatus*.** The structural and functional features of HD proteins were studied on the basis of the amino acid sequences of the homeodomain proteins encoded by the *matA* locus of *P. ostreatus* strains PC9 and PC15, whose genomes were completely sequenced and published on the DOE JGI website (Joint Genome Institute, http://genome.jgi.doe.gov/). To predict the presence of signal sequences, the SignalP 4.1 (www.cbs.dtu.dk/services/SignalP) and PSORT (k-Nearest Neighbors Classifier algorithm) software were used [14]. To predict the protein structure (globular or transmembrane, as well as the presence of transmembrane domains and surface loops) on the basis of the hydrophilicity/hydrophobicity of amino acid sequences, the Kyte Doolittle Hydropathy Plot software (http://gcat.davidson.edu/DGPB/kd/kyte-doolittle.htm) was used [15]. To predict the structural organization of the protein molecule (secondary structure), the SWISS-MODEL Secondary Structure Prediction and Domain Assignment software (http://swissmodel.expasy.org/workspace/index.php) was used [16].

## RESULTS AND DISCUSSION

### Quantitative Analysis of Alleles of Sexual Compatibility Loci in Natural Strains of Oyster Mushroom Pleurotus ostreatus

For five dikaryotic natural isolates (M-8, M-9, M-13, M-14, and M-17), monokaryotic (haploid) tester strains were obtained (four testers for each natural strain), and mon–mon crosses with the sexual compatibility testers (strain M-38) of *P. ostreatus* were carried out (Table 1). In all mon–mon crosses, the sexual compatibility of the monokaryotic tester strains obtained from the natural dikaryotic strains with the testers of the *P. ostreatus* M-38 strain was demonstrated. Sexual compatibility was determined by the presence of characteristic clamps on the mycelium at the sites of contact of monokaryotic (haploid) mycelia, which indicated the dikaryotization of monokaryons. The formation of clamps in the septum region is a good and convenient diagnostic sign of dikaryotic mycelium in many homobasidial fungi, including the members of the genus *Pleurotus*. In our experiment, sexual compatibility with all four testers of the M-38 strain indicated that the crossed natural strains had different alleles of the A and B loci of sexual compatibility (Table 1). The presence of different alleles in five natural strains (M-8, M-9, M-13, M-14, and M-17) was also confirmed in mutual crosses of monokaryotic testers of natural dikaryotic strains with each other in all possible combinations (Table 2, results for three dikaryotic strains are presented). Strains M-8 and M‑9 were heteroallelic at both mating compati-bility loci (*matA* and *matB*), whereas strains M-8 and M-13 had the same alleles of the B locus (50% of compatible combinations in mon–mon crosses). Strains M‑14 and M-17 were sexually compatible with each other and as well as with strains M-8, M-9, and M-13 (data not shown in Table 2). The remaining 12 natural dikaryotic strains were tested in di–mon crosses, i.e., in crosses of dikaryons with monokaryotic testers of *P. ostrestus* strain M-38 (Table 1). In the case of sexually compatible combinations in the di–mon crosses, dikaryotization of the monokaryotic tester occurred, which was accompanied by the formation of clamps on the monokaryotic mycelium.

**Table 1.** Allele frequency distribution of mating type loci among *Pleurotus ostreatus* natural isolates as determined by mon–mon and di–mon crossing

Natural isolates				Haploid testers of *P. ostreatus* M-38			
				m1*	m2	m3	m4
				A1B1**	*A1B2*	*A2B1*	*A2B2*
Mon–mon mating between monokaryotic tester strains of natural isolates	Strain М-8	m1	*A3B3*	+***	+	+	+
		m2	*A3B4*	+	+	+	+
		m3	*A4B3*	+	+	+	+
		m4	*A4B4*	+	+	+	+
	Strain М-9	m1	*A5B5*	+	+	+	+
		m2	*A5B6*	+	+	+	+
		m3	*A6B5*	+	+	+	+
		m4	*A6B6*	+	+	+	+
	Strain М-13	m1	*A7B7*	+	+	+	+
		m2	*A7B8*	+	+	+	+
		m3	*A8B7*	+	+	+	+
		m4	*A8B8*	+	+	+	+
	Strain М-14	m1	*A9B9*	+	+	+	+
		m2	*A9B10*	+	+	+	+
		m3	*A10B9*	+	+	+	+
		m4	*A10B10*	+	+	+	+
	Strain М-17	m1	*A11B11*	+	+	+	+
		m2	*A11B12*	+	+	+	+
		m3	*A12B11*	+	+	+	+
		m4	*A12B12*	+	+	+	+
Di–mon mating	Dikaryotic strains		М-2	+	+	+	+
			М-3	+	+	+	+
			М-4	+	+	+	+
			М-5	+	+	+	+
			М-7	+	+	+	+
			М-15	+	+	+	+
			М-16	+	+	+	+
			М-18	+	+	+	+
			М-19	+	+	+	+
			М-20	+	+	+	+
			М-21	+	+	+	+
			М-22	+	+	+	+

*m, monokaryotic tester strain; **AxBy, alleles of mating type loci; ***+, mating compatibility (dikaryotization and clamp connections developing).

**Table 2.** Mon-mon crossing between tester strains derived from *Pleurotus ostreatus* natural isolates

Monokaryotic tester strains			Strain М-8				Strain М-9				Strain М-13			
			m1	m2	m3	m4	m1	m2	m3	m4	m1	m2	m3	m4
			*A3B3*	*A3B4*	*A4B3*	*A4B4*	*A5B5*	*A5B6*	*A6B5*	*A6B6*	*A7B3*	*A7B4*	*A8B3*	*A8B4*
Strain М-8	m1	*A3B3*	–	–	–	+*	+	+	+	+				
	m2	*A3B4*	–	–	+	–**	+	+	+	+				
	m3	*A4B3*	–	+	–	–	+	+	+	+				
	m4	*A4B4*	+	–	–	–	+	+	+	+				
Strain М-9	m1	*A5B5*	+	+	+	+	–	–	–	+				
	m2	*A5B6*	+	+	+	+	–	–	+	–				
	m3	*A6B5*	+	+	+	+	–	+	–	–				
	m4	*A6B6*	+	+	+	+	+	–	–	–				
Strain М-13	m1	*A7B3*	–	+	–	+	+	+	+	+	–	–	–	+
	m2	*A7B4*	+	–	+	–	+	+	+	+	–	–	+	–
	m3	*A8B3*	–	+	–	+	+	+	+	+	–	+	–	–
	m4	*A8B4*	+	–	+	–	+	+	+	+	+	–	–	–

*Sexual compatibility (dikaryotization and clamp connection developing); **sexual incompatibility. Strains M-8 and M-9 are heteroallelic at both mating type loci; strains М-8 and М-13 have the same alleles at the *B* locus.

Thus, in the analysis of 17 natural isolates of *P. ostreatus*, at least ten alleles of the *matA* locus and 8 alleles of the *matB* locus were found, which is consistent with the previously obtained data on the multiallelicity of the sexual compatibility loci in our studies and in the research by other authors [3, 5, 17]. Sivolapova et al. [18] mapped the *mat* loci of *P. ostreatus* and showed that the *matA* locus is located on chromosome III, and the *matB* locus is located on chromosome IX.

### Analysis of the Structure of the Mata Locus of Sexual Compatibility in Species of the Genus Pleurotus

Analysis of the structure of the *matA* locus of sexual compatibility was performed for three species of the genus *Pleurotus* (*P. ostreatus*, *P. eryngii*, and *P. djamor*) on the basis of the data for partially or fully sequenced genomes. Previously, a detailed structural analysis of the *matA* locus in the model fungus *Coprinus cinerea* showed that this locus encodes the HD1 and HD2 homeodomain proteins, which are transcription factors; however, these proteins significantly differ in structure and amino acid sequences [7, 19]. Both classes of homeodomain proteins contain three α‑helical regions, the third of which includes the conserved WFXNXR DNA-binding motif; the sequences of the DNA-binding domain also differ. For example, the HD2 type exhibits a high DNA-binding activity, whereas HD1 possesses a weaker activity but has nuclear localization signals and an activatory domain [7]. *C. cinerea* class HD2 proteins have a DNA-binding domain length of 60 aa, which is typical for homeodomains, whereas the HD1 homeodomains contain three additional amino acid residues between the first and second α-helices. The N termini of protein sequences are of particular importance in the structure of HD1 and HD2 homeodomain proteins, because they function as dimerization domains between the compatible HD1 and HD2 proteins. Only the HD1/HD2 heterodimer can function as a transcription factor and is capable to regulate the expression of morphogenesis genes during the development of a fertile dikaryon followed by maturation of fruiting bodies [19, 20].

**Fig. 1.**
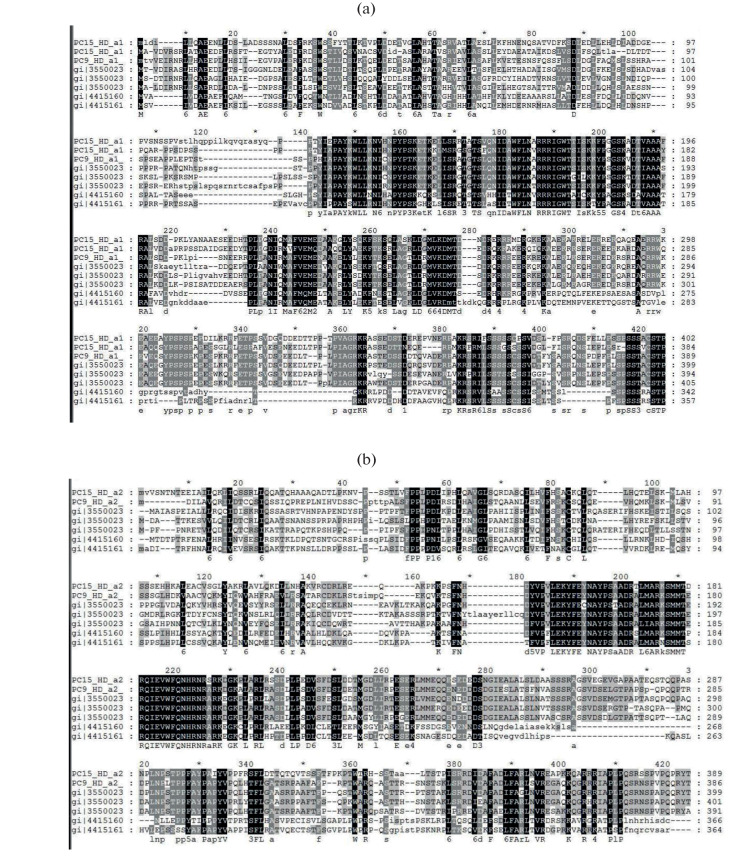
Fragment of amino acid sequence alignment of HD proteins between strains PC15 *Pleurotus ostreatus*, PC9 *P. ostreatus*; strains CCMSSC00488 *P. eryngii*, CCMSSC00489 *P. eryngii*; and strains RV95/134.104 *P. djamor* and RV95/957.30 *P. djamor*: (a) class HD1 proteins; (b) class HD2 proteins. The Dialign 2.2.1 software; rectangles highlight the conserved motif WFXNXR.

In our study, we found no homologous regions between the *hd* gene sequences in the homokaryotic (haploid) strains of three oyster mushroom species (*Pleurotus ostreatus*, *P. eryngii*, and *P. djamor*) at the nucleotide level, that indicates a high degree of variability in the sequences of these genes. Homologies between the sequences of homeodomain proteins were found solely at the amino acid level. As a result of the alignment of the amino acid sequences of HD1 and HD2 proteins, we found highly variable domains concentrated at the N terminus of the protein molecule, as well as homology domains. Two classes of transcription factors HD1 and HD2 were differed not only in the amino acid sequences, but also in length. The HD1 protein sequences were generally 40–50 aa residues longer than the HD2 protein sequences. Particularly variable regions in the proteins of classes HD1 and HD2 were due to insertions and deletions. Such variable regions presumably belong to the looped spatial structures of the protein. Both classes of homeodomain sequences of *P. ostreatus*, *P. eryngii*, and *P. djamor* had the conserved WFXNXR motif (in the region of 120–190 aa in the HD1 protein and in the region of 140–195 aa in the HD2 protein; Fig. 1). This conserved WFXNXR motif, as mentioned above, is involved in the direct binding of the HD1/HD2 regulatory heterodimeric protein to the DNA molecule (DNA-binding motif). It is also of interest that, in addition to the WFXNXR motif, another conserved motif, HNPYPT/S, was found in the HD1 proteins of three oyster mushroom species, which was absent in the HD2 proteins. Thus, the results of in silico analysis showed the structural similarity of the homeodomain proteins of two classes (HD1 and HD2) in three species of the genus *Pleurotus* (*P. ostreatus*, *P. eryngii*, and *P. djamor*): the presence of conserved motifs of DNA-binding domains and highly variable dimerization domains at the N terminus of protein molecules. Importantly, the dimerization of HD1–HD2 homeodomain proteins is possible only between proteins from different sexual partners (i.e., when different alleles of the *matA* locus interact in crossing). Therefore, it is not surprising that the N termini as dimerization sites significantly differ in sequences between the allelic variants of proteins from different sexual partners. It is believed that significant variability in the N‑terminal amino acid sequences of homeodomain proteins ensures the existence of multiple alleles and variants of allelic interactions between the fungus haploid partners in crosses [21].

**Fig. 2.**
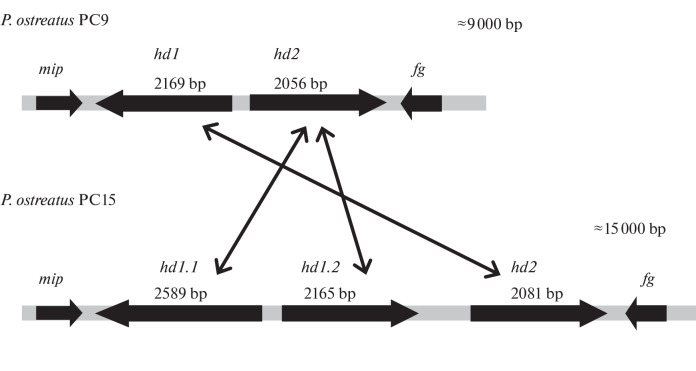
Structure of the *matA* locus of sexual compatibility of *Pleurotus ostreatus* and the scheme of allelic interactions between homeodomain proteins. The *mip* gene encodes a mitochondrial intermediate peptidase; the *fg* (beta-flanking protein) gene encodes a protein with an unknown function; *hd* genes encode homeodomain proteins.

A more detailed analysis of the *matA* locus structure was performed for two homokaryotic *P. ostreatus* strains, PC9 and PC15. It should be noted that PC9 and PC15 strains were obtained from the same dikaryotic parent, the original strain N001, by dedicaryotization (splitting up the dikaryon into homokaryons) in Larraya’s laboratory [22]. Despite the origin from the same parental dikaryotic strain N001, our analysis in silico  showed an extremely divergent structure of the *matA* locus in these homokaryon strains: the *matA* locus of strain PC9 is represented by one copy of the *hd1* gene and one copy of the *hd2* gene, whereas the *matA* locus of strain PC15 has two *hd1* copies, *hd1.1* and *hd1.2* (class HD1 proteins) and one *hd2* copy (class HD2 proteins) (Fig. 2).

In other words, the homokaryotic strain PC9 is characterized by the standard (canonical) structure of the *matA* locus, i.e., contains a pair of genes (*hd1* and *hd2*) encoding HD1 and HD2 proteins, whose transcription is regulated from a common intergenic promoter region. A similar structure of the *matA* locus (the presence of two genes, *PDa1* and *PDa2*, encoding homeodomain transcription factors HD1 and HD2, respectively) was described for the species *P. djamor* [6]. The homokaryotic strain PC15 is distinguished by the presence of an additional (second) copy of the gene encoding the class HD1 protein, as a result of which the *matA* locus of this strain contains three copies of the *hd* genes (*hd1.1*, *hd1.2*, and *hd2*) (Fig. 2). It is difficult to predict from sequencing data which of the copies of the genes encoding the class HD1 proteins is active in the PC15 strain, because the copies of the genes (*hd1.1*  and *hd1.2*) are transcribed in opposite directions, although they have a common promoter region. It can be assumed that one of the copies of the *hd1* gene appeared as a result of duplication or insertion. The likelihood of occurrence of insertions and deletions within the *matA* locus of basidiomycetes has already been described earlier [23]. In general, the high variability of nucleotide sequences of *hd* genes, which is believed to be associated with the mechanism of accumulation of point mutations, is a tool for achieving a greater allelic diversity of loci, and multiallelicity is ultimately aimed at reducing the likelihood of inbreeding in natural populations of basidiomycetes. As in other basidiomycetes, the highly variable *matA* locus in the studied strains PC9 and PC15 of *P. ostreatus* is flanked by more conserved sequences encoding the *mip* gene (mitochondrial intermediate peptidase) and the *fg* gene with an unknown function (beta-flanking protein) (Fig. 2).

### Analysis of the HD1 and HD2 Amino Acid Sequences of Pleurotus ostreatus Proteins

When analyzing the amino acid sequences of the HD1 and HD2 proteins of *P. ostreatus* on the basis of multiple alignments of the sequences of homeodomain proteins, we estimated the probabilistic localization of the studied class HD1 proteins in the cell: for the HD1.1 protein (strain PC15), 73.9% nuclear localization, 17.4% cytoskeletal localization, 4.3% secretory system vesicles, and 4.3% membrane localization; for the HD1.2 protein (strain PC15), 52.2% nuclear localization, 26.1% cytoplasmic localization, 17.4% cytoskeletal localization, and 4.3% secretory system vesicles; for HD1 protein (strain PC9), 73.9% nuclear localization, 17.4% cytoplasmic localization, and 8.7% cytoskeletal localization. For the class HD2 proteins, the following probabilistic localization in the cell was predicted: for the HD2 protein (strain PC15), 65.2% nuclear localization, 26.1% cytoplasmic localization, and 8.7% cytoskeletal localization; for the HD2 protein (strain PC9), 82.6% nuclear localization, 8.7% cytoskeletal localization, 4.3% cytoplasmic localization, and 4.3% secretory system vesicles. Thus, for the studied homeodomain proteins of both classes (HD1 and HD2), the probabilistic nuclear localization was shown. It should be noted that the program searched for the nuclear localization signal sequences by the sites enriched either in arginine (R) and lysine (K) or in proline (P) and histidine (H), which are markers of nuclear localization  signal sequences. Another feature is the fact that representatives of the class HD2 proteins have characteristic motifs of the DNA-binding homeobox domain: MARKSMMTDRQIEVWFQNHRNSR for the HD2 protein of the PC15 strain (starting from 179 aa) and MARKSMMTERQIEVWFQNHRNRAR for the HD2 protein of the PC9 strain (starting from 177 aa) (graphical data are not shown). However, no homeobox motifs were found within the HD1 protein sequences. Therefore, it can be assumed that the DNA-binding domain of the class HD2 proteins is more conserved. On the basis on the assessment of the hydrophilicity/hydrophobicity of amino acid residues in HD proteins using the Kyte Doolittle Hydropathy Plot software, it was shown that the homeodomain proteins of HD1 and HD2 classes lack the transmembrane hydrophobic regions; therefore, the proteins have a globular structure, which is characteristic of transcription factors (in this article, data are not presented in detail). The globular structure of the proteins and the absence of transmembrane signal sequences were also confirmed using the SignalP 4.1 software. The characteristic WFXNXR DNA-binding domain in class HD1 proteins is at position 125–175 aa, whereas in class HD2 proteins it is at position 145–200 aa; these regions are positionally homologous to the corresponding sequences of homeodomain proteins of the model fungus *Coprinus cinerea* [24]. It should be noted that, in general, class HD2 proteins of *Pleurotus ostreatus* had a more conserved structure of the DNA-binding homeodomain.

In this work, we demonstrated the extremely divergent structure of the *matA* locus of sexual compatibility in two sexually compatible homokaryotic strains (PC9 and PC15) of *P. ostreatus*: the *matA* locus of the PC9 strain is represented by one copy of the *hd1* gene and one copy of the *hd2* gene, whereas the *matA* locus of the PC15 strain has two copies of *hd1.1* and *hd1.2* and one copy of the *hd2* gene. The in silico analysis of the amino acid sequences of the HD1 and HD2 homeodomain proteins showed that all the studied HD proteins have a globular structure and nuclear localization. All HD sequences had a variable N-terminus and a more conserved DNA-binding domain with a characteristic conserved WFXNXR motif in the third α-helix. As shown earlier [7, 19], the N-terminal sequences in the structure of HD1 and HD2 homeodomain proteins function as dimerization domains between the compatible HD1 and HD2 partner proteins. Due to this function, the N terminus is characterized by a high degree of amino acid sequence variability between different alleles of the sexual compatibility locus. An active HD1/HD2 heterodimer can form only between the partner proteins HD1 and HD2, which are transcribed from different alleles of the *matA* locus in the dikaryon formed between the sexually compatible homokaryotic strains. Our results of analysis of the structural organization of the *matA* locus and the homeodomain proteins encoded by this locus in *P. ostreatus* support the standpoint that HD proteins function as transcription factors and, therefore, have nuclear localization and DNA-binding motifs. The results of this study indicate a high structural variability of the sequences of homeodomain proteins. The multiallelicity of the *matA* locus is achieved both due to the different copy number of coding genes within the locus and due to the variability of the coding gene sequences. Another interesting feature is that the HD1 and HD2 proteins of tetrapolar basidiomycetes are homologous to the homeodomain proteins Matα2 and Mata1, which are the main regulatory units of the unilocus (bipolar) system of sexual reproduction in the ascomycetes *Saccharomyces cerevisiae* [19, 23]. Therefore, the study of the possible mechanisms of genetic sex determination in fungi is relevant and will help to understand the complex issues of the evolution of sexual reproduction systems. The issues of the transition from functional heterothallism to homothallism in fungi, from the bipolar system of sexual compatibility to tetrapolar, and vice versa, as well as the return to pseudohomothallism, which is observed in some groups of fungi and which is often due to parasitic lifestyle, still remain largely unresolved. However, it is hoped that further accumulation of data on fungal genome sequencing will undoubtedly shed light on the evolution of genetic sex determination systems and understanding the key mechanisms for achieving biological diversity, including the allelic diversity.
